# Comparison of senescence progression in mesenchymal cells from human umbilical cord walls measured by immunofluorescence and flow cytometry of p16 and p21

**DOI:** 10.31744/einstein_journal/2020AO5236

**Published:** 2020-10-09

**Authors:** Aline da Silva, Carla de Azevedo Piccinato, Luiz Roberto Sardinha, Thiago Pinheiro Arrais Aloia, Anna Carla Goldberg

**Affiliations:** 1 Hospital Israelita Albert Einstein São PauloSP Brazil Hospital Israelita Albert Einstein, São Paulo, SP, Brazil.

**Keywords:** p16, Cyclin-dependent kinase inhibitor p21, Mesenchymal stem cells, Cellular senescence, Immunofluorescence, Flow cytometry, Western blotting

## Abstract

**Objective:**

To follow the expansion of mesenchymal stem cells from umbilical cords by two classic senescence markers, p16 (INK4A) and p21 (CDKN1A), using practical, fast, and less expensive methods than the gold standard Western blotting technique, to evaluate its applicability in the laboratory.

**Methods:**

Mesenchymal stem cells from umbilical cords were isolated from Wharton’s jelly and, after quality control, morphological and immunophenotypic characterization by flow cytometry, were expanded in culture until coming close to cell cycle arrest (replicative senescence).

**Results:**

A comparison was made between young cells, at passage 5, and pre-senescent cells, at passage 10, evaluating the protein expression of the classic cell senescence markers p16 and p21, comparing the results obtained by Western blotting with those obtained by flow cytometry and indirect immunofluorescence.

**Conclusion:**

Follow-up of cell cultures, through indirect p16 immunofluorescence, allows the identification of mesenchymal stem cells from umbilical cord cultures at risk of reaching replicative senescence.

## INTRODUCTION

Mesenchymal stem cells (MSC), initially described in 1968, are clonogenic cells capable of self-renewal and differentiation into other types of cells.^(1)^ This differentiation into adipocytes and into the osteogenic and chondrogenic lineages can be reproduced *in vitro*. The MSC can also be expanded in cultures and tested in regenerative medicine protocols, potentially becoming useful for cell therapy.^(2)^

A rich source of MSC is the umbilical cord because the connective tissue that involves umbilical vessels, called Wharton’s jelly, contains cells considered an alternative to the use of MSC from bone marrow and other tissues. This is due to its easy isolation, its greater potential to expand when in a culture,^(3)^ and its greater accessibility, with few ethical restrictions. Additionally, as very young (neonatal) cells, they are considered to have undergone less environmental interference from infections and health-damaging products or suffered the consequence of aging.^(4)^

With the advancement of the body’s age, the MSCs, just as any other adult cells, can become senescent. In senescence, the cellular cycle is arrested, interrupting cell division; however, the cells remain alive, but are dysfunctional.^(5)^

The term “replicative senescence” was coined by Hayflick, in 1960, when he showed that human diploid fibroblasts had a limited capacity to proliferate *in vitro*, since there was reduction in the length of telomeres and arrested cell division. However, the cells still remained alive and secreted metabolites.^(6,7)^ Later, it was shown that mitotically normal cells can also enter senescence by the action of stressors,^(8)^ such as damage to DNA, which accompanied by the increase in poorly folded proteins and oxidative stress^(9)^ caused an irreversible loss of the proliferative ability.^(8,10)^ It is agreed that this state of senescence protects the organism, impeding the cell from an abnormal growth, thus avoiding appearance of cells with tumorigenic potential.^(11)^ Therefore, induction of senescence results directly from signaling systems activated during the cellular cycle. To regulate this induction there are control proteins that activate and others that inhibit their targets with opposing results. Within the group of positive control proteins are the cyclin-dependent kinases (CDK), which trigger signaling cascades that lead to the arrest in cell proliferation. Because of this action, they are known as tumor suppressors. This is the case of p16 (P16INK4 gene or CDKN2, cyclin-dependent kinase inhibitor 2A) and p21 (CDKN1A, cyclin-dependent kinase inhibitor 1A) proteins. Even though characterization of senescence is still based on the presence or absence of a group of robust markers,^(12)^ accumulation of p16 is considered a key marker of cellular senescence. Furthermore, p21 is also a tumor suppressor protein and its expression can be utilized as a marker of young cells with a high proliferative capacity, contrasting with p16, which is increasingly expressed in later passages.^(13)^

The evaluation of cellular senescence markers is commonly done by Western blotting (WB), considered the gold standard for evaluation of protein expression. However, it is a laborious technique requiring several steps and needs a large number of cells. For this reason, measuring senescence usually implies in ending the culture to obtain the necessary quantity of cells. Therefore, we decided to evaluate if other reliable methods present sensitivity to measure cell senescence, carrying out a comparative analysis with WB. An assessment of senescence in cell cultures was done using indirect immunofluorescence (IFI) and flow cytometry, techniques that are faster and use a smaller number of cells. Additionally, these techniques offer the possibility of storing remaining cells or even of maintaining ongoing experiments. Finally, the possibility of cell storage and even of further cell expansion is important when the goal is the use in cell therapy. Our hypothesis was that one of these techniques could be used instead of WB, allowing evaluation of senescence during the experiments, without, however, interrupting the cell culture.

## OBJECTIVE

To compare the measurements of p16 and p21 proteins by immunofluorescence and/or flow cytometry with Western blotting, at p5 and p10 passages, that is, at two timepoints of the mesenchymal stem cell culture, to monitor the progression of senescence in stages prior to the cell cycle arrest and to determine the feasibility of the two alternative techniques.

## METHODS

### Obtaining the cord

Human umbilical cords (n=4) were harvested after obtaining Informed Consent, according to ethical criteria established by the National Health Council. The protocol was approved under CAAE: 17079113.4.0000.0071 and #353.781. Healthy samples were those that followed the evaluation criteria of the Public Umbilical Cord Blood Bank of the *Hospital Israelita Albert Einstein* (HIAE): pregnant women aged over 18 years, with gestations equal to or greater than 35 weeks, in whom water did not break more than 18 hours before, who attended to at least two appointments during pregnancy, with no infection or fever at birth, and delivery by cesarean section. Before delivery and after maternal blood collections, serology was performed to confirm absence of hepatitis A, B, and C, HIV I and II, HTLV I and II, cytomegalovirus, toxoplasmosis, Chagas’ disease, and syphilis, besides hemoglobin electrophoresis *Agência Nacional de Vigilância Sanitária/Resolução da Diretoria Colegiada* (ANVISA/RDC 153/2004). Samples were harvested as of 2013 and the study was concluded in June 2018.

### Cell isolation and culture

After blood removal, cords were processed at our laboratory within four hours of harvesting, according to the protocol published by Paladino et al.,^(14)^ Cells from the umbilical cord wall were sown onto 25 or 75cm^2^ culture flasks (Corning, St. Louis, MO) containing Dulbecco’s Modified Eagle’s Medium (DMEM-LG) supplemented with 20% fetal bovine serum (FBS), 1% antibiotic-antifungal (penicillin 100 units/mL, streptomycin 100μg/mL, amphotericin 250ng/mL, and L-glutamine 2mM/mL) solution and maintained in a humidified incubator, with 5% carbon dioxide, at 37^o^C. The cells were stored in liquid nitrogen at passage 3. All reagents were acquired from Gibco^®^ (New Grand Island, USA) except where specified. We used two aliquots to obtain cells at passage 5 (p5) and a third sample from the same cord for expansion until passage 10 (p10). After thawing, samples were cultured in the same medium, but adding only 10% of SFB. A total of 4,000 cells/cm^2^ were sown and culture medium was changed every 48 hours. Cell passaging was done at 70% confluence, utilizing a 1% collagenase solution for five minutes. Cells were characterized as MSC at passage 4 as per criteria established by the International Society for Cellular Therapy (ISCT)^(15)^ and maintained in culture until passage 10. Human Embryonic Kidney 293 (HEK-293) cells were used as positive control and MCF7 (Mammary Gland, Breast; Derived from Metastatic Site: Pleural Effusion) cells as negative control for p16 analysis, both obtained from the Rio de Janeiro Cell Bank (Rio de Janeiro, Brazil). The same cells worked respectively as negative and positive controls for p21.

### Sterility, mycoplasma detection, and microbiological testing of cell cultures

After 48 hours, 750µl of supernatant were collected for microbiological analysis to confirm absence of microorganisms in the cell culture. This analysis was performed by the Clinical Laboratory of the *Hospital Israelita Albert Einstein* using the Ziehl-Neelsen stain for high-resistance bacteria, direct observation for fungi, in addition to bacterioscopic analysis. *Mycoplasma* detection was done by Enzyme-Linked Immunosorbent Assay (ELISA) using anti-*Mycoplasma* antibodies. After checking for absence of microorganisms, samples were either frozen or maintained in culture for future assays.

### Immunophenotypic characterization of mesenchymal stem cells at passage 3 and/or 4

According to ISCT, MSC must exhibit a specific profile of cell surface markers, positive for CD105, CD73, CD44, CD29, CD166, and CD90, and negative for hematopoietic markers (CD14, CD34, CD45, CD117, CD133), endothelial markers (CD31, CD106, CD133 ) and cell surface HLA-DR molecules.^(16)^ There are three minimum requirements for the classification of a MSC population. The first is its isolation through selective adherence, in culture, to the plastic surface; the second is a set of positive and negative markers analyzed by flow cytometry; and the third is the capacity of the cells to differentiate into osteocytes, adipocytes, and chondrocytes. The characterization of the cells in this study was carried out by Paladino et al.^(14)^

### Western blotting

Lysate proteins were quantified by the Pierce method (BCA protein Assay Kit, Thermo Fisher, USA) and separated by electrophoresis in a 12% polyacrylamide gel for 1 hour 20 minutes, at 100 V, in neutral pH buffer containing SDS (sodium dodecyl sulfate) at 1% (Invitrogen, USA). After the run, the proteins were transferred to Amersham nitrocellulose (GE Healthcare Life Sciences, USA) membranes using a 20% methanol buffer (Merck KGaA, Darmstadt, Germany) for 1 hour, at 100V. The membranes were then incubated in 5% bovine serum albumin (BSA, Cell Signaling, USA) blocking solution for 1 hour followed by the primary p21 (1:1000 – Ab109520, Abcam, USA) or p16 (1:1000 – Ab108349, Abcam, USA) antibodies in BSA solution, overnight. Subsequently, membranes were washed three times for five minutes with TBS-T (Tris-HCl, 24.23g, NaCl 80.06g, and 0.1% Tween^®^ 20) solution and then, incubated with the secondary antibody conjugated to horseradish peroxidase (HRP) (1:2000, Cell Signaling, USA) diluted in TBS-T + 2% Molico milk (Nestlé, Brazil), for 1 hour, in a dark environment, under gentle shaking. After another three washes, membranes received ECL Prime Western Blotting System developer solution (GE Healthcare Life Sciences, USA). The chemiluminescent solution was analyzed in a Chemidoc reader (Bio-Rad, Hercules, USA) revealing the presence of the specific protein band. Analysis was done by the ImageLab program (Biorad, Hercules, USA). Each test was done in duplicate.

### Indirect immunofluorescence

Cells in culture were transferred to circular glass cover slips fitted into 24-well plates, sown at a concentration of 4,000/cm^2^ and cultured until reaching 50% to 70% confluence. Culture medium was then removed, cells were washed with phosphate buffered saline (PBS) four times and fixed with 4% paraformaldehyde, for 10 minutes. Next, cells were washed with PBS and cell membranes permeabilized with a Triton-X 100 (non-ionic detergent, Sigma-Aldrich, USA) solution at 0.01%, diluted in PBS, for 15 minutes. Following four washes with PBS, at 5-minute intervals, nonspecific bonds were blocked with BSA at 1%, for 30 minutes. After repeating PBS washes, cells were incubated for 24 hours with the primary p16 (1:100 - Ab108349, *rabbit anti-human*, Abcam, USA) or p21 (1:100 - Ab109520, *rabbit anti-human*, Abcam, USA) antibodies, in a dark and humid environment at 4°C. After 24 hours, cells were washed three times with PBS at 5-minute intervals, and then, incubated with the secondary antibody. We used the secondary antibody diluted in 1% BSA to detect p16 (1:400, *rabbit anti-human* IgG (H+L), F(ab’)2 Fragment, Alexa Fluor^®^ 555 Conjugate, Cell Signaling, USA) or p21, (*rabbit anti-human* IgG (H+L), F(ab’)2 Fragment, AlexaFluor^®^ 488 Conjugate, Cell Signaling, USA), for 30 minutes, maintaining the membrane in room temperature under gentle shaking and protected from light. Next, cells were washed with PBS, in 5-minute intervals; the last wash was done with distilled water and immediately after, cover slips were recovered from their wells. Cover slips were maintained protected from light at room temperature until completely dry, for staining with a DAPI (VECTASHIELD Antifade Mounting Medium with DAPI, Vector Laboratories, EUA) reagent. Slides were analyzed on a confocal fluorescence microscope (Zeiss confocal LSM 880, Germany), and cell count was carried out on ten fields, in triplicate, regardless of the number of cells in each field. Mean fluorescence intensity was calculated correcting by the number of cells counted. Fluorescence was measured using the ZEN lite program (Zeiss, Germany).

### Flow cytometry

Cultured cells were recovered, washed with PBS 1X containing 1% BSA and centrifuged at 400g, for five minutes. To stain for p16 we utilized 2x10^5^ cells/tube fixed with 4% paraformaldehyde, for 10 minutes, at room temperature. The test was done in triplicate, as in the case of IFI. After permeabilization in PBS 1X + 0.1% Tween^®^ 20 (Merck KGaA, Darmstadt, Germany) for 20 minutes, at 4°C, cells were washed with PBS containing 0.1% Tween^®^ 20, at room temperature. The cell suspension was then centrifuged at 400g for five minutes. To avoid nonspecific binding, we added BSA 5% + 0.1% Tween^®^ 20, for 15 minutes, at 4°C. Cells were washed once again and centrifuged for intracellular staining with *rabbit anti-human* CDKN2A/p16INK4A (1:100, clone: EPR1473, Abcam, USA) monoclonal antibody. After incubation for 30 minutes at 4°C, washing and centrifugation were repeated twice. Cells were then incubated with an ***r****abbit anti-human* polyclonal secondary antibody conjugated with PE (1:100, clone: Poly4064, Biolegend, USA), washed and suspended in PBS + 1% BSA for analysis in a BD FACSARIA (Becton Dickinson, San Jose, CA) flow cytometer. A protocol similar to the one used for p21 was used to stain for p16, except for fixing cells in 80% methanol at 4°C, followed by incubation for 20 minutes at 4°C and *rabbit anti-human* p21 monoclonal antibody (1:100, clone: EPR 362, Abcam, USA) for the intracellular staining. Results were analyzed with FlowJo software (Treestar, Ashland, Oregon), considering mean fluorescence intensity (MFI) values after counting 2x10^5^ cells/tube.

### Experimental design

**Figure 1:**
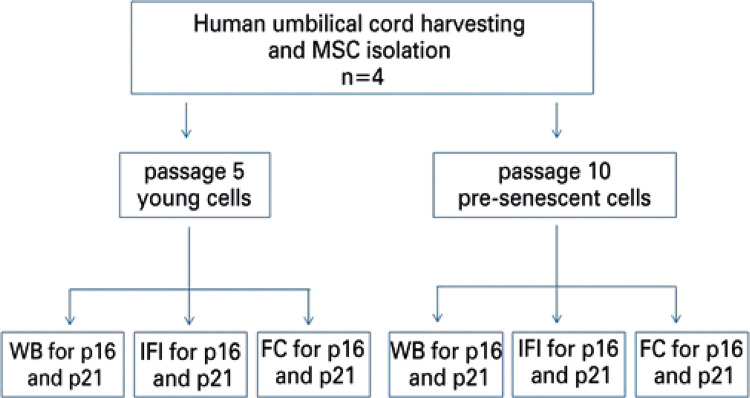
Experimental design MSC: mesenchymal stem cells; WB: Western blotting; IFI: indirect immunofluorescence; FC: flow cytometry; p: passage.

Figure 1 shows the experimental design used in this study. It is important to point out that the comparison between the different methods was enabled by the concomitant use of the same cell cultures in the three methods of choice. Thus, when dealing with primary cultures, and not with established lineages, this experimental design sought to enable the detection of low albeit normal, levels of proliferation of cells in a culture eventually harboring more senescent cells, and therefore, with decreased proliferation rates. The complete experiment was repeated with four different cells, and the results are only qualitatively described.

## RESULTS

### Western blotting showed the heterogeneity of individual progression to replicative senescence

**Figure 2:**
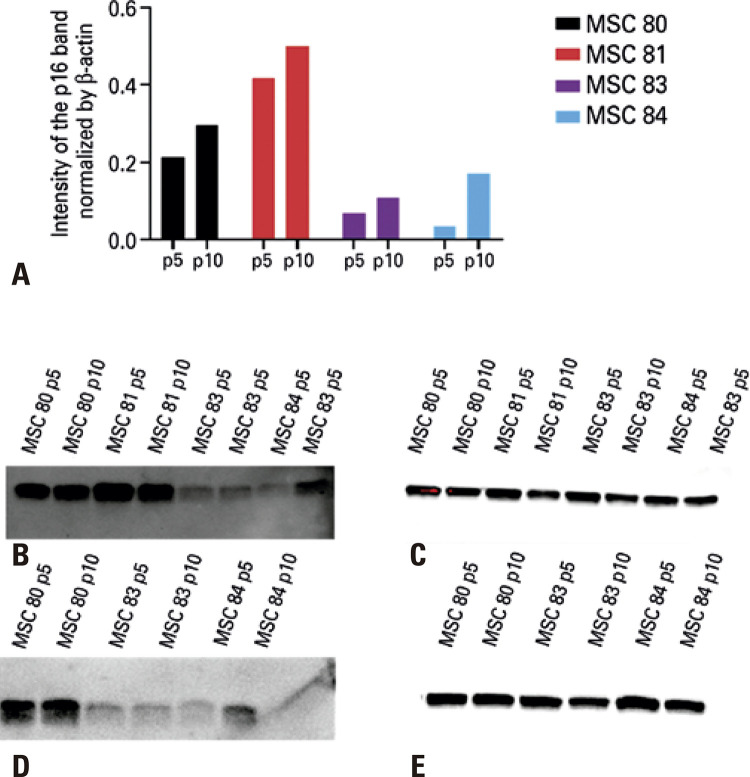
Western blotting of p16 in mesenchymal stem cells (n=4) at passage 5 and passage 10. (A) intensity of the p16 band normalized by the beta-actin band for each sample. The vertical bar indicates variation in measurements. (B) image of p16 bands at passage 5. (C) image of beta-actin bands at passage 5. (D) image of p16 bands at passage 10. (E) image of beta-actin bands at passage 10 p: passage; MSC: mesenchymal stem cell.

**Figure 3:**
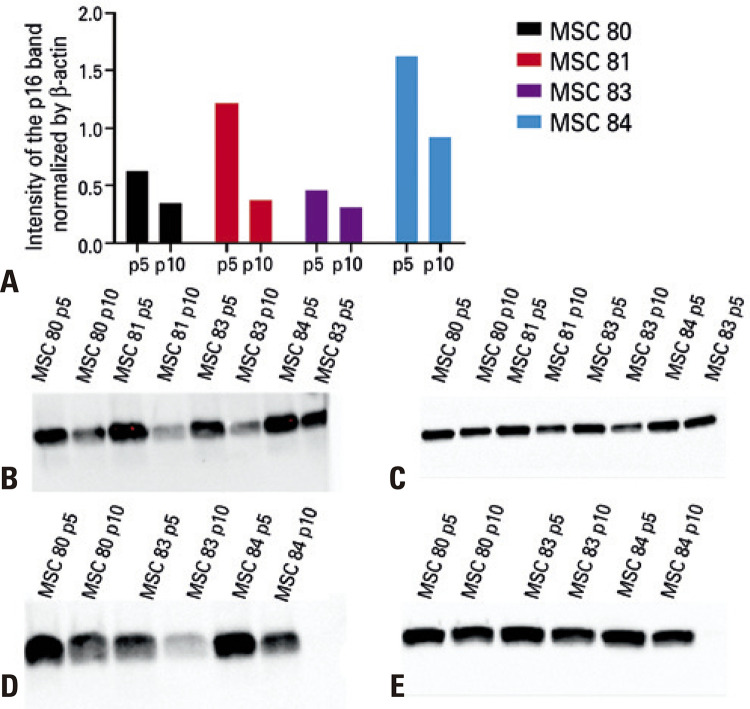
Western blotting of p16 in mesenchymal stem cells (n=4) at passage 5 and passage 10. (A) intensity of the p21 band normalized by the beta-actin band for each sample. The vertical bar indicates the variation in measurements. (B) image of p21 bands at passage 5. (C) image of beta-actin bands at passage 5. (D) image of p21 bands at passage 10. (E) image of beta-actin bands at passage 10 P: passage; MSC: mesenchymal stem cell.

As expected, using the WB technique, p16 and p21 levels showed the progression towards replicative senescence. Measurement of p16 and p21 by WB displayed an increase in p16 and a decrease in p21 at passage 10 compared to passage 5 (Figures 2 and 3, respectively), consistent with the expression previously observed in senescent mesenchymal cells from other sources^(17)^ and with the heterogeneous profile observed in the different cords studied.^(14)^ Each MSC culture showed typical morphologic changes, with a distinct pattern of population doubling (Supplementary figure 1A), but with the predicted increase in cytoplasm (Supplementary figure 1B).

**Figure:**
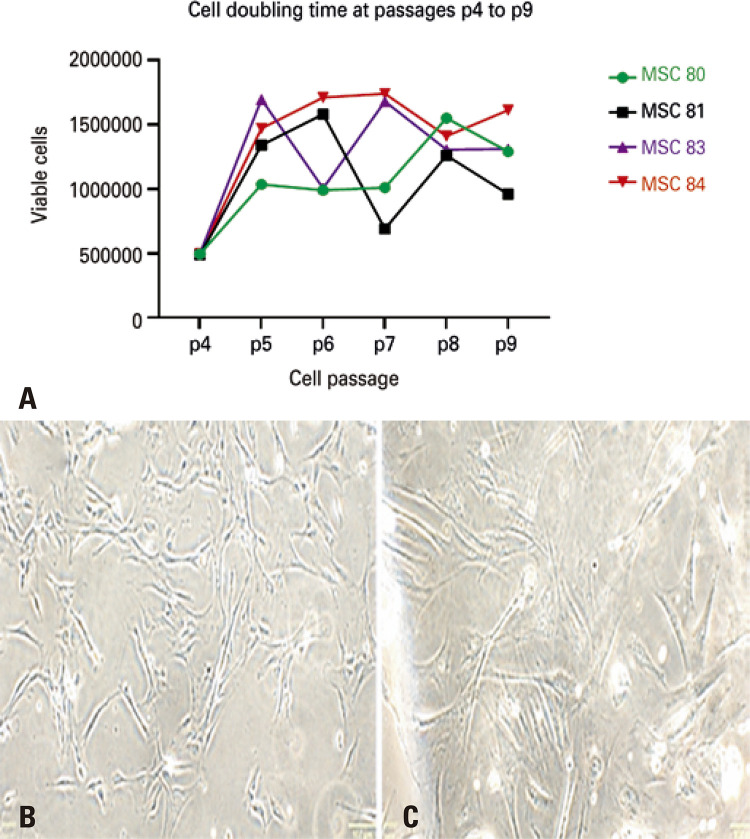
Supplementary figure 1. (A) Optical microscopy image of mesenchymal stem cells isolated from cord 81. Optical microscopy image of mesenchymal stem cell 81 in (B) passage 5 and (C) passage 10, showing cells with increased volume and greater refringence, indicating the progression to replicative senescence MSC: mesenchymal stem cell; p: passage.

### Monitoring of p16, but not of p21 by immunofluorescence shows a pattern comparable to Western blotting

As a semiquantitative method, IFI permits follow-up of cultures, but no quantitative measurements, such as those obtained by WB. Nevertheless, expression of p16 at passage 10 was increased in all the samples analyzed by IFI, with a profile comparable to the variation observed with WB (Figures 4A and 4B).

**Figure 4A:**
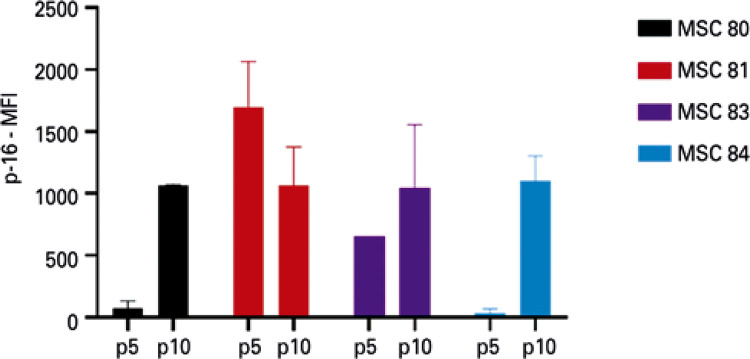
Mean fluorescence intensity of p16 in mesenchymal stem cells (n=4) at passage 5 and passage 10, analyzed by indirect immunofluorescence. The vertical bar indicates the variation in measurements p: passage; MFI: mean fluorescence intensity; MSC: mesenchymal stem cell.

**Figure 4B:**
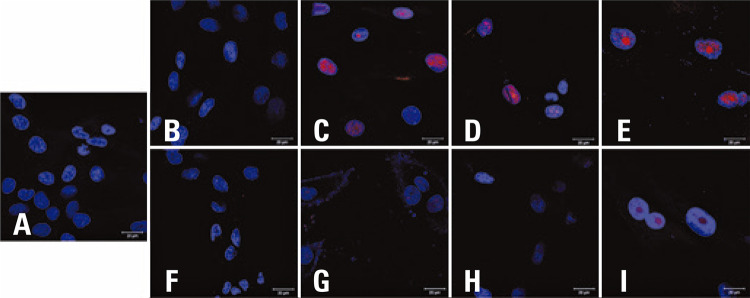
Fluorescence intensity of p16 (in red) in the mesenchymal stem cells (n=4) at passage 5 and passage 10, analyzed by indirect immunofluorescence. Nuclei stained with DAPI (in blue). (A) negative control using secondary antibody; (B) mesenchymal stem cell 80 at p5; (C) mesenchymal stem cell 80 at p10; (D) mesenchymal stem cell 81 at p5; (E) mesenchymal stem cell 81 at p10; (F) mesenchymal stem cell 83 at p5, (G) mesenchymal stem cell 83 at p10; (H) mesenchymal stem cell 84 at p5; (I) mesenchymal stem cell 84 at p10

On the other hand, IFI of p21 has not proven to be a reliable marker in comparison to WB. Only two of the four cells showed the same behavior, with greater expression of p21 at passage 5 relative to passage 10 (Supplementary figures 2 and 3).

**Figure:**
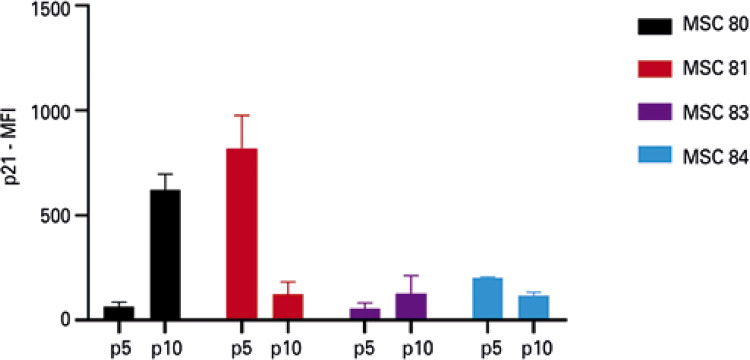
Supplementary figure 2. Mean fluorescence intensity of p21 in the mesenchymal stem cells (n=4) at passage 5 and passage 10, analyzed by indirect immunofluorecence. The vertical bar indicates variation in measurements p: passage; MFI: mean fluorescence intensity; MSC: mesenchymal stem cell.

**Figure:**
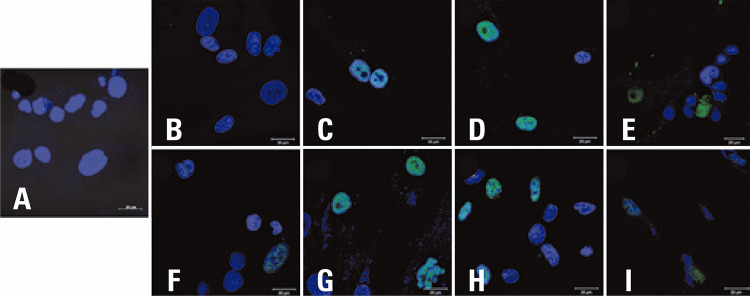
Supplementary figure 3. Fluorescence intensity of p21 (in green) in mesenchymal stem cells (n=4) at passage 5 and passage 10, analyzed by indirect immunofluorescence. Nuclei stained with DAPI (in blue). (A) negative control using secondary antibody; (B) mesenchymal stem cell 80 at p5; (C) mesenchymal stem cell 80 at p10; (D) mesenchymal stem cell 81 at p5; (E) mesenchymal stem cell 81 at p10; (F) mesenchymal stem cell 83 at p5; (G) mesenchymal stem cell 83 at p10; (H) mesenchymal stem cell 84 at p5; (I) mesenchymal stem cell 84 at p10

### Monitoring p21, but not p16, by flow cytometry is comparable to Western blotting

**Figure 5:**
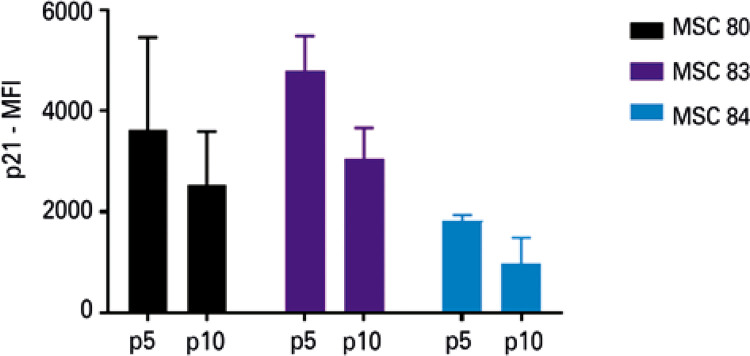
Mean fluorescence intensity of p21 in the mesenchymal stem cells (n=4) at passage 5 and passage 10, analyzed by flow cytometry. The vertical bar indicates the variation in measurements p: passage; MFI: mean fluorescence intensity; MSC: mesenchymal stem cell.

When we compared flow cytometry with WB, it was possible to observe that expression of p21 by flow cytometry and WB presented a similar behavior (Figure 5)*.* On the other hand, the expression of p16 did not show the expected results (Supplementary figure 4). Additionally, since the quantity of cells necessary for analysis by flow cytometry was greater than for IFI, a fourth sample, which exhibited a very low proliferation rate at passage 10 could not be analyzed.

**Figure:**
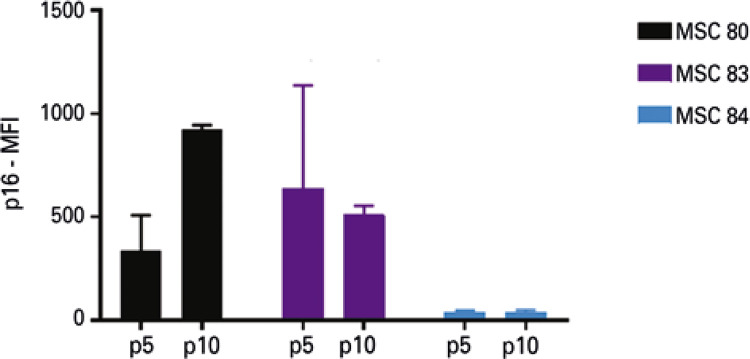
Supplementary figure 4. Mean fluorescence intensity of p16 in mesenchymal stem cells (n=4) at passage 5 and passage 10, analyzed by flow cytometry. The vertical band indicates variation in measurements p: passage; MFI: mean fluorescence intensity; MSC: mesenchymal stem cell.

## DISCUSSION

To make a proper comparison of the two techniques, IFI and flow cytometry, with WB cells from the same culture of each of the cords were used, and comparisons were done simultaneously. Although still a preliminary study, results indicated that monitoring p16 levels on IFI slides cultured in parallel with each sample are an interesting approach to monitoring MSC cultures. These cultures are long lasting, with a significant number of passages needed to achieve the necessary expansion in cell numbers, and the concomitant analysis of the evolution of the culture helps to identify those MSC with a higher risk of entering replicative senescence.

We also analyzed p21, but the results show that even when using flow cytometry, with a data profile comparable to the gold standard WB, the need for more cells extracted directly from the culture, and not from a cover slip cultured in parallel (as in the case of IFI), makes the practice more difficult. Furthermore, it has already been described, in other cells, that p21 levels decrease gradually while p16 levels increase.^(18,19)^ This is probably due to the fact that the functional control of p21 occurs primarily through phosphorylation and intracellular compartmentalization during the different phases of the cell cycle. Several weeks after human fibroblast cultures reach senescence, the p21 level continues to diminish gradually as the p16 level increases.^(18,19)^ These findings, and the fact that p21 is not induced in senescent p53-deficient cells that have a prolonged replicative life,^(20,21)^ suggest that p53 initiates the inhibition of this cellular replication, at least in part, by inducing p21. The subsequent increase in p16 then acts to maintain the arrest in cell growth, leading to senescence. Thus, even though p21 is a marker of cellular aging, monitoring it ends up being more complex and out of the scope of our study. In contrast, the observation of p21 levels by flow cytometry, using antibodies that also measure the degree of protein phosphorylation, may render monitoring of progression to cellular senescence with p21 useful for research.

There are clear limitations to our study, since only four samples were tested of a single tissue type and it would be important to compare them to other MSC, for example, from bone marrow and adipose tissue, to validate this very practical strategy of assessing the progression of senescence in cultures. Traditionally, cells have their validation in terms of quality, cell markers, and proliferative capacity made at low passages (passages 4 or 5), and, in the final phases before therapeutic application, only microbiological and cytogenetic controls are carried out. The degree of senescence, that is, the cell capacity for proliferation is not evaluated before their use. Thus, we believe that it is possible to follow the intracellular increase of p16 during cell expansion with the purpose of cell therapy. This follow-up can be useful to improve the evaluation of the effectiveness of donor cells, which, together with the factors inherent to recipients, such as age, and type and severity of the underlying disease impact the success of therapy with MSC.

## CONCLUSION

Follow-up of cell cultures using indirect immunofluorescence of p16, allows identifying mesenchymal stem cell cultures at risk of reaching replicative senescence.
