# Supplementary material for: Comparison of senescence progression in mesenchymal cells from human umbilical cord walls measured by immunofluorescence and flow cytometry of p16 and p21
Source: Einstein (Sao Paulo). 2020 Oct 9;18:eAO5236. doi: 10.31744/einstein_journal/2020AO5236 (PMC7546682; doi:10.31744/einstein_journal/2020AO5236)
Supplement: Translation

# Translation: Comparação da progressão da senescência em células mesenquimais de parede de cordão umbilical humano medida por imunofluorescência e citometria de fluxo de p16 e p21

**Authors:** Aline da Silva, Carla de Azevedo Piccinato, Luiz Roberto Sardinha, Thiago Pinheiro Arrais Aloia, Anna Carla Goldberg

**Affiliations:** 1 Brasil Hospital Israelita Albert Einstein, São Paulo, SP, Brasil.

**Keywords:** p16, Inibidor de quinase dependente de ciclina p21, Células-tronco mesenquimais, Senescência celular, Imunofluorescência, Citometria de fluxo, Western blotting

## Abstract

**Objetivo:**

Acompanhar a expansão de células-tronco mesenquimais de cordão umbilical por dois marcadores clássicos de senescência, p16 (INK4A) e p21 (CDKN1A), usando métodos práticos, rápidos e com custo menor do que a técnica padrão-ouro de *Western blotting*, para avaliar sua aplicabilidade em laboratório.

**Métodos:**

Células-tronco mesenquimais de cordão umbilical foram isoladas da geleia de Wharton e, após controle de qualidade e caracterização morfológica e imunofenotípica por citometria de fluxo, foram expandidas em cultura, até chegarem próximas à parada do ciclo celular (senescência replicativa).

**Resultados:**

Foi feita a comparação entre células jovens, na passagem 5, e células pré-senescentes, na passagem 10, avaliando a expressão proteica dos marcadores clássicos de senescência celular p16 e p21, comparando os resultados obtidos por *Western blotting* com os obtidos por citometria de fluxo e imunofluorescência indireta.

**Conclusão:**

O seguimento de culturas celulares, por meio da imunofluorescência indireta de p16, permite identificar as culturas de células-tronco mesenquimais de cordão umbilical em risco de atingirem a senescência replicativa.

## INTRODUÇÃO

As células-tronco mesenquimais (CTM), descritas inicialmente em 1968, são células clonogênicas, capazes de autorrenovação e de diferenciação em outros tipos de células.^(1)^ Essa diferenciação em adipócitos e nas linhagens osteogênica e condrogênica pode ser reproduzida *in vitro*. As CTM também podem ser expandidas em cultura e testadas em protocolos de medicina regenerativa, tornando-se potencialmente úteis para terapia celular.^(2)^

Uma fonte rica em CTM é o cordão umbilical, pois o tecido conjuntivo que envolve os vasos umbilicais, chamado de geleia de Wharton, contém células consideradas uma alternativa ao uso de CTM de medula óssea e de outros tecidos, por sua facilidade de isolamento, seu maior potencial de expansão em cultura^(3)^ e sua maior acessibilidade, com poucas restrições éticas. Além disso, por serem células de idade muito jovem (neonatal), considera-se terem sofrido menor interferência ambiental de infecções e produtos danosos à saúde, ou ação pelo envelhecimento.^(4)^

Com o avanço da idade do organismo, as CTMs, como quaisquer outras células adultas, podem entrar em senescência. Na senescência, ocorre a parada do ciclo celular, interrompendo a divisão celular; entretanto as células continuam vivas, mas disfuncionais.^(5)^

O termo “senescência replicativa” foi cunhado em 1960 por Hayflick quando mostrou que fibroblastos diploides humanos tinham capacidade limitada para proliferar *in vitro*, pois ocorriam a redução do comprimento dos telômeros e a parada da divisão celular. Entretanto as células mantinham-se ainda vivas e secretando metabólitos.^(6,7)^ Depois, foi mostrado que células mitoticamente normais também podem entrar em senescência pela ação de estressores,^(8)^ como danos ao DNA que, acompanhados do aumento de proteínas mal dobradas e do estresse oxidativo,^(9)^ acarretam uma perda irreversível da capacidade proliferativa.^(8,10)^ É consenso que esse estado de senescência protege o organismo, impedindo a célula de ter um crescimento anormal e evitando o aparecimento de células com potencial tumorigênico.^(11)^ Assim, a indução da senescência decorre diretamente de sistemas de sinalização ativados durante o ciclo celular. Para regular essa indução, existem proteínas controladoras positivas que ativam, e outras negativas que inibem seus alvos e contrapõem suas ações. Dentro do grupo de proteínas positivas, estão as cinases dependentes de ciclina (CDK), que desencadeiam cascatas de sinalização, as quais levam à parada de proliferação celular. Por essa ação, são conhecidas como supressoras de tumores. É o caso das proteínas p16 (gene P16INK4 ou CDKN2 - *cyclin-dependent kinase inhibitor 2A*) e p21 (CDKN1A - *cyclin-dependent kinase inhibitor 1A*). Apesar da caracterização da senescência ainda se basear na presença ou na ausência de um grupo de marcadores robustos,^(12)^ o acúmulo de p16 tem sido considerado um marcador-chave da senescência celular. A p21 também é uma proteína supressora de tumor, sendo que seu nível de expressão pode ser utilizado como marcador de células jovens, com alta capacidade proliferativa, contrastando com p16, expressa em nível elevado em passagens tardias.^(13)^

A avaliação de marcadores de senescência celular é comumente realizada pela técnica de *Western blotting* (WB), considerada padrão-ouro para avaliação de expressão proteica. Entretanto é uma técnica trabalhosa, por demandar várias etapas, e que necessita de um número grande de células. Por esse motivo, usualmente, analisar um ensaio de senescência implica no término da cultura para a obtenção da quantidade necessária de células. Assim, propusemos-nos testar outros métodos sensíveis e com resultados confiáveis no quesito de avaliação da senescência celular, efetuando uma análise comparativa com os resultados obtidos por WB. Foi feita uma avaliação da senescência das culturas celulares por imunofluorescência (IFI) indireta e citometria de fluxo, técnicas mais rápidas e que utilizam número menor de células. Além disso, essas técnicas oferecem a possibilidade de armazenamento das células restantes ou mesmo a continuação dos experimentos. Finalmente, a possibilidade de armazenar mais células e mesmo de continuar sua expansão é importante quando a meta é usá-las em terapia celular. Nossa hipótese foi a de que uma dessas técnicas poderia ser empregada ao invés de WB, permitindo a avaliação de senescência durante os experimentos sem, todavia, interromper a cultura.

## OBJETIVO

Comparar medidas das proteínas p16 e p21 por imunofluorescência e/ou citometria de fluxo com *Western blotting* nas passagens p5 e p10, ou seja, em dois momentos da cultura de células-tronco mesenquimais, para monitorar a progressão da senescência em uma fase anterior à parada do ciclo celular, e determinar a viabilidade das duas técnicas alternativas.

## MÉTODOS

### Obtenção do cordão

Cordões umbilicais humanos (n=4) foram coletados, após a obtenção do Termo de Consentimento Livre e Esclarecido, conforme critérios éticos estabelecidos pelo Conselho Nacional de Saúde. O protocolo aprovado recebeu o CAAE: 17079113.4.0000.0071 e parecer 353.781. Foram consideradas amostras saudáveis as que obedeceram aos critérios de avaliação do Banco Público de Sangue de Cordão Umbilical do Hospital Israelita Albert Einstein (HIAE): gestantes com idade acima de 18 anos; com tempo de gravidez igual ou superior a 35 semanas; cuja bolsa não poderia ter se rompido há mais de 18 horas; que realizaram no mínimo duas consultas durante a gravidez; sem infecção ou febre no momento do parto; e parto por cesárea. Antes do parto, após a coleta do sangue da mãe, foram realizados exames sorológicos, para confirmar a ausência de vírus da hepatite A, B e C, HIV I e II, HTLV I e II, citomegalovírus, toxoplasmose, doença de Chagas e sífilis, além da eletroforese de hemoglobina (Agência Nacional de Vigilância Sanitária/Resolução da Diretoria Colegiada - ANVISA/RDC 153/2004). As amostras foram coletadas a partir de 2013 e o estudo finalizado em junho de 2018.

### Isolamento e cultivo das células

Após a retirada do sangue, os cordões foram processados em nosso laboratório em até 4 horas após a coleta, de acordo com o protocolo publicado por Paladino et al.^(14)^ As células da parede de cordão umbilical foram semeadas em frascos de cultura 25 ou 75cm^2^ (Corning, St. Louis, MO) contendo *Dulbecco’s Modified Eagle’s Medium* (DMEM-LG) suplementado com 20% soro fetal bovino (SFB), 1% antibiótico-antimicótico (penicilina 100 unidades/mL, estreptomicina 100μg/mL, anfotericina 250ng/mL e L-glutamina 2mM/mL) e mantidas em incubadora umidificada, com 5% de dióxido de carbono, a 37^o^C. As células foram armazenadas em nitrogênio líquido na passagem 3. Todos os reagentes, quando não explicitado, foram adquiridos de Gibco^®^ (New Grand Island, EUA). Para obtenção das células na passagem 5 (p5) foram utilizadas duas amostras, e, para a expansão até a passagem 10 (p10), foi utilizada uma terceira amostra, obtida do mesmo cordão. Após o descongelamento, a cultura de todas as amostras passou a ser feita com o mesmo meio, mas com apenas 10% de SFB adicionado. Foram semeadas 4.000 células/cm^2^. O meio de cultura foi trocado a cada 48 horas. A passagem das células foi feita na confluência de 70%, utilizando solução de colagenase a 1% por 5 minutos. Na passagem 4, as células foram caracterizadas como CTM segundo os critérios estabelecidos pela *International Society for Cellular Therapy* (ISCT),^(15)^ e o cultivo foi mantido até a passagem 10. Para o controle positivo na análise de p16, foi utilizada a linhagem HEK-293 (*Human Embryonic Kidney* 293), e, para o controle negativo, foi utilizada a linhagem MCF7 (*Mammary Gland, Breast; Derived from Metastatic Site: Pleural Effusion*), ambas obtidas do Banco de Células do Rio de Janeiro (Rio de Janeiro, Brasil). As mesmas células funcionaram como controles negativo e positivo para p21, respectivamente.

### Esterilidade e detecção de micoplasma e exame microbiológico na cultura

Para a confirmação da ausência de microrganismos na cultura celular, após 48 horas, 750µl do sobrenadante foram coletados para análise microbiológica. A análise foi efetuada pelo Laboratório Clínico do Hospital Israelita Albert Einstein utilizando os métodos de Ziehl-Neelsen para bactérias de alta resistência, observação direta para fungos, além da análise bacterioscópica. A detecção de *Mycoplasma* foi feita por *Enzyme-Linked Immunosorbent Assay* (ELISA) usando anticorpos anti-*Mycoplasma*. Depois de certificada a ausência de microrganismos, as amostras foram congeladas ou mantidas em cultura para desenvolver os experimentos.

### Caracterização imunofenotípica de células-tronco mesenquimais na passagem 3 e/ou 4

Segundo a ISCT, as CTM devem exibir perfil específico de expressão na superfície celular, que é positivo para CD105, CD73, CD44, CD29, CD166 e CD90, e negativo para marcadores hematopoiéticos (CD14, CD34, CD45, CD117, CD133), marcadores endoteliais (CD31, CD106, CD133 ) e molécula de superfície HLA-DR.^(16)^ São três as exigências mínimas para classificar uma população de CTM. A primeira é sua separação com base em sua aderência seletiva, em cultura, à superfície do plástico; a segunda é de um conjunto de marcadores positivos e negativos analisados por citometria de fluxo; e a terceira é a capacidade de as células se diferenciarem em osteócitos, adipócitos e condrócitos. Essa caracterização foi feita nas células desse estudo por Paladino et al.^(14)^

### Western blotting

As proteínas do lisado foram quantificadas pelo método de Pierce (BCA protein Assay Kit, Thermo Fisher, EUA) e separadas por eletroforese em gel de poliacrilamida 12%, por 1 hora 20 minutos, a 100V, em tampão de pH neutro contendo SDS (dodecil sulfato de sódio) a 1% (Invitrogen, EUA). Após a corrida, as proteínas contidas no gel foram transferidas para membranas de nitrocelulose Amersham (GE *Healthcare Life Sciences*, EUA) em tampão 20% metanol (Merck KGaA, Darmstadt, Alemanha) por 1 hora, a 100V. As membranas foram incubadas em solução de bloqueio 5% albumina sérica bovina (BSA, Cell Signaling, EUA) por 1 hora e, em seguida, incubadas com o anticorpo primário p21 (1:1000 – Ab109520, Abcam, EUA) ou p16 (1:1000 – Ab108349, Abcam, EUA) em solução de BSA, por uma noite. Em seguida, a membrana foi lavada três vezes por 5 minutos com a solução de TBS-T (Tris-HCl, 24,23g, 80,06g NaCl e 0,1% Tween^®^ 20) e, em seguida, incubada com o anticorpo secundário conjugado com peroxidase de rábano (HRP) (1:2000, Cell Signaling, EUA) diluído em TBS-T + leite Molico 2% (Nestlé, Brasil), por 1 hora, em ambiente escuro, sob agitação lenta. Após nova lavagem por três vezes, a membrana recebeu a solução reveladora ECL *Prime Western Blotting System* (GE *Healthcare Life Sciences*, EUA). O produto quimioluminescente foi analisado no leitor Chemidoc (Bio-Rad, Hercules, EUA), revelando presença da banda proteica específica. A mensuração foi feita pelo programa *Image Lab* (Biorad, Hercules, EUA). Cada teste foi feito em duplicata.

### Imunofluorescência indireta

Células em cultura foram transferidas para lamínulas circulares de vidro contidas em placas de 24 poços, semeadas na concentração de 4.000/cm^2^ e cultivadas até atingirem a confluência de 50% a 70%. Em seguida, foi retirado o meio de cultura, e as células foram lavadas com tampão fosfato-salina (PBS), por quatro vezes e fixadas com paraformaldeído 4%, por 10 minutos. Em seguida, as células foram lavadas com PBS e as membranas celulares, permeabilizadas com a solução de Triton-X 100 (detergente não iônico, Sigma-Aldrich, EUA) a 0,01%, diluído em PBS, por 15 minutos. Após lavagem com PBS, quatro vezes, em intervalos de 5 minutos, foi feito, nas células, o bloqueio de ligações inespecíficas com BSA a 1%, por 30 minutos. Após repetir a lavagem com PBS, as células foram incubadas por 24 horas com os anticorpos primários p16 (1:100 – Ab108349, *rabbit anti-human* Abcam, USA) ou p21 (1:100 –Ab109520, *rabbit anti-human*, Abcam, USA), mantidas em ambiente escurecido e úmido a 4°C. Após 24 horas, as células foram lavadas com PBS em intervalos de 5 minutos por três vezes e, em seguida, incubadas com o anticorpo secundário. Para a detecção do p16, utilizamos o anticorpo secundário diluído em BSA 1% (1:400, *rabbit anti-human* IgG (H+L), F(ab’)2 Fragment, Alexa Fluor^®^ 555 Conjugate, Cell Signaling, EUA) e para p21 (*rabbit anti-human* IgG (H+L), F(ab’)2 Fragment, Alexa Fluor^®^ 488 Conjugate, Cell Signaling, EUA), por 30 minutos, mantendo a membrana sob agitação leve, em temperatura ambiente e protegida da luz. Logo depois, as células foram lavadas com PBS, em intervalos de 5 minutos; a última lavagem das células fixadas foi feita com água destilada, e, imediatamente, as lamínulas dos poços foram recuperadas. As lamínulas foram mantidas protegidas da luz e em temperatura ambiente, até secarem por completo para receber a solução do reagente DAPI (VECTASHIELD *Antifade Mounting Medium with* DAPI, Vector Laboratories, USA). As lâminas foram analisadas em um microscópio confocal de fluorescência (Zeiss confocal LSM 880, Alemanha), sendo feita a contagem das células em dez campos, em triplicata, independentemente do número de células. Calculou-se a média de intensidade de fluorescência (MIF) corrigida pelo número de células contadas. A fluorescência foi mensurada usando o programa ZEN lite (Zeiss, Alemanha).

### Citometria de fluxo

Células em cultura foram recuperadas, lavadas com PBS 1X contendo BSA 1% e centrifugadas a 400g, por 5 minutos. Para a marcação da proteína p16, utilizamos 2x10^5^ células/tubo, fixada com paraformaldeído a 4%, por 10 minutos, em temperatura ambiente. O teste foi realizado em triplicata, como no caso da IFI. Após a permeabilização celular incubando as células em PBS 1X + 0,1% Tween^®^ 20 (Merck KGaA, Darmstadt, Alemanha) por 20 minutos, a 4°C, foi feita lavagem adicionando novamente PBS contendo 0,1% Tween^®^ 20, em temperatura ambiente. A suspensão de células foi, então, centrifugada a 400g, por 5 minutos. Para evitar ligações inespecíficas, adicionamos BSA 5% + 0,1% Tween^®^ 20, por 15 minutos, a 4°C. As células foram lavadas novamente e centrifugadas para a marcação intracelular com o anticorpo monoclonal *rabbit anti-human* CDKN2A/p16INK4A (1:100, clone: EPR1473, Abcam, EUA). Após incubação por 30 minutos a 4°C, foram feitas a lavagem e a centrifugação, por duas vezes. Utilizamos o anticorpo secundário policlonal *rabbit anti-human* conjugado com PE (1:100, clone: Poly4064, Biolegend, USA), e as células foram incubadas, com lavagem e suspensão em PBS + 1% BSA para leitura no citômetro de fluxo BD FACSARIA (Becton Dickinson, San Jose, CA). Para a proteína p21, utilizamos o protocolo similar à marcação da proteína p16. A diferença residiu na fixação das células em metanol a 80%, a 4°C, seguida de incubação por 20 minutos a 4°C. Para essa marcação intracelular, utilizamos o anticorpo monoclonal *rabbit anti-human* p21 (1:100, clone: EPR 362, Abcam, EUA). Para análise dos resultados, foi utilizado o programa FlowJo (Treestar, Ashland, Oregon), sendo considerados os valores da MIF após contagem de 2x10^5^ células/tubo.

### Desenho experimental

A figura 1 mostra o desenho experimental empregado nesse estudo. É importante salientar que a comparação dos diversos métodos foi possibilitada pelo emprego concomitante das mesmas culturas celulares nos três métodos de escolha. Assim, em se tratando de culturas primárias, e não de linhagens já estabelecidas, esse desenho experimental visou possibilitar diferenciar uma taxa de proliferação baixa, mas normal, das células de uma possível cultura com células mais senescentes e, assim, com taxa de proliferação diminuída. O experimento completo foi repetido com quatro células diferentes, e os resultados estão descritos apenas qualitativamente.

**Figura 1:** Desenho experimental CTM: células-tronco mesenquimais; WB: *Western blotting;* IFI: imunofluorescência indireta; CF: citometria de fluxo; p: passagem.

## RESULTADOS

### *Western blotting* mostrou a heterogeneidade da progressão individual para senescência replicativa

Como esperado, usando a técnica de WB, as proteínas p16 e p21 mostraram a progressão para a senescência replicativa. A mensuração das proteínas p16 e p21 por WB mostrou aumento de p16 e a diminuição de p21 na passagem 10 comparada à passagem 5 (Figuras 2 e 3, respectivamente), condizente com a expressão já observada em células mesenquimais senescentes de outras origens^(17)^ e com o perfil heterogêneo nos diferentes cordões estudados.^(14)^ Cada cultura de CTM apresenta alterações morfológicas típicas, com padrão de dobramento populacional distinto (Figura suplementar 1A), mas com o aumento citoplasmático (Figura suplementar 1B) previsto.

**Figura 2:** *Western blotting* da proteína p16 nas células-tronco mesenquimais (n=4) em passagem 5 e passagem 10. (A) intensidade da banda de p16 normalizada pela intensidade da banda de beta-actina para cada amostra. A barra vertical indica a variação nas medidas. (B) imagem das bandas de p16 na passagem 5. (C) imagem das bandas de beta-actina na passagem 5. (D) imagem das bandas de p16 na passagem 10. (E) imagem das bandas de beta-actina na passagem 10 p: passagem; CTM: célula-tronco mesenquimal.

**Figura 3:** *Western blotting* da proteína p21 nas células-tronco mesenquimais (n=4) em passagem 5 e passagem 10. (A) intensidade da banda de p21 normalizada pela intensidade da banda de beta-actina para cada amostra. A barra vertical indica a variação nas medidas. (B) imagem das bandas de p21 na passagem 5. (C) imagem das bandas de beta-actina na passagem 5. (D) imagem das bandas de p21 na passagem 10. (E) imagem das bandas de beta-actina na passagem 10 P: passagem; CTM: célula-tronco mesenquimal.

**Figure:** Figura suplementar 1. (A) Imagem por microscopia ótica de células isoladas de cordão de célula-tronco mesenquimal 81. Imagem por microscopia ótica de célula-tronco mesenquimal 81 em (B) passagem 5 e (C) passagem 10, mostrando células com volume aumentado e maior refringência, indicando a progressão para a senescência replicativa CTM: célula-tronco mesenquimal; p: passagem.

### Monitoramento por imunofluorescência de p16, mas não de p21, mostra padrão comparável ao *Western blotting*

A IFI, sendo um método semiquantitativo, foi adequado para acompanhamento de culturas, e não para medidas quantitativas, como as obtidas por WB. No entanto, a expressão da proteína p16 em passagem 10 analisada pela IFI estava aumentada em todas as amostras, com perfil de variação comparável ao obtido com WB (Figuras 4A e 4B).

**Figura 4A:** Intensidade média de fluorescência da proteína p16 nas células-tronco mesenquimais (n=4) em passagem 5 e passagem 10, analisadas por imunofluorescência indireta. A barra vertical indica a variação nas medidas p: passagem; MIF: média de intensidade de fluorescência; CTM: célula-tronco mesenquimal.

**Figura 4B:** Intensidade de fluorescência da proteína p16 (em vermelho) nas células-tronco mesenquimais (n=4) em passagem 5 e passagem 10, analisadas por imunofluorescência indireta. Marcação dos núcleos com DAPI (em azul). (A) controle negativo usando anticorpo secundário; (B) célula-tronco mesenquimal 80 em p5; (C) célula-tronco mesenquimal 80 em p10; (D) célula-tronco mesenquimal 81 em p5; (E) célula-tronco mesenquimal 81 em p10; (F) célula-tronco mesenquimal 83 em p5, (G) célula-tronco mesenquimal 83 em p10; (H) célula-tronco mesenquimal 84 em p5; (I) célula-tronco mesenquimal 84 em p10

Por outro lado, a IFI da proteína p21 não demonstrou ser marcador fidedigno em comparação ao WB*.* Apenas duas das quatro células mostraram o mesmo comportamento, com a expressão da p21 maior na passagem 5 em relação à passagem 10 (Figura suplementar 2 e 3).

**Figure:** Figura suplementar 2. Intensidade média de fluorescência da proteína p21 nas células-tronco mesenquimais (n=4) em passagem 5 e passagem 10, analisadas por imunofluorescência indireta. A barra vertical indica a variação nas medidas p: passagem; MIF: média de intensidade de fluorescência; CTM: célula-tronco mesenquimal.

**Figure:** Figura suplementar 3. Intensidade de fluorescência da proteína p21 (em verde) nas células-tronco mesenquimais (n=4) em passagem 5 e passagem 10, analisadas por imunofluorescência indireta. Marcação dos núcleos com DAPI (em azul). (A) controle negativo usando anticorpo secundário; (B) célula-tronco mesenquimal 80 em p5; (C) célula-tronco mesenquimal 80 em p10; (D) célula-tronco mesenquimal 81 em p5; (E) célula-tronco mesenquimal 81 em p10; (F) célula-tronco mesenquimal 83 em p5; (G) célula-tronco mesenquimal 83 em p10; (H) célula-tronco mesenquimal 84 em p5; (I) célula-tronco mesenquimal 84 em p10

### Monitoramento por citometria de fluxo de p21, mas não de p16, é comparável ao *Western blotting*

Comparando a técnica de citometria de fluxo com WB, foi possível observar que a expressão da proteína p21 por citometria de fluxo apresentou comportamento semelhante (Figura 5) à análise feita por WB*.* Por outro lado, a expressão de p16 não mostrou os resultados esperados (Figura suplementar 4). Além disso, como a quantidade de células necessárias para a análise por citometria de fluxo foi maior do que para IFI, uma quarta amostra, que apresentou taxa de proliferação na passagem 10 já muito baixa, não permitiu que essa análise fosse feita.

**Figura 5:** Intensidade média de fluorescência da proteína p21 nas células-tronco mesenquimais (n=4) em passagem 5 e passagem 10, analisadas por citometria de fluxo. A barra vertical indica a variação nas medidas p: passagem; MIF: média de intensidade de fluorescência; CTM: célula-tronco mesenquimal.

**Figure:** Figura suplementar 4. Intensidade média de fluorescência da proteína p16 nas células-tronco mesenquimais (n=4) em passagem 5 e passagem 10, analisadas por citometria de fluxo. A barra vertical indica a variação nas medidas p: passagem; MIF: média de intensidade de fluorescência; CTM: célula-tronco mesenquimal.

## DISCUSSÃO

Para efetuarmos uma comparação adequada das duas técnicas, IFI e citometria de fluxo, com WB, foram utilizadas células da mesma cultura de cada um dos cordões, e as comparações foram efetuadas simultaneamente. Apesar de ser ainda um estudo preliminar, os resultados indicaram que fazer o acompanhamento dos níveis de p16 em lâminas de IFI cultivadas em paralelo com cada amostra é uma abordagem interessante de seguimento de culturas de CTM. Essas culturas são de longa duração, com número significativo de passagens para alcançar a expansão necessária no número de células, e a análise em paralelo da evolução da cultura permite identificar aquelas CTM com maior risco de entrar em senescência replicativa.

Analisamos também a proteína p21, mas os resultados mostram que, mesmo na citometria de fluxo, o perfil dos dados seja comparável ao padrão-ouro WB, a necessidade de maior número de células extraídas diretamente da cultura, e não de uma lamínula cultivada em paralelo (no caso da IFI), torna a prática mais dificultada. Além disso, já foi descrito, em outras células, que os níveis de p21 diminuem gradualmente, à medida que os níveis de p16 aumentam.^(18,19)^ Isso provavelmente decorre do fato do controle funcional dessa proteína se dar primordialmente pelo nível de fosforilação e pela compartimentalização intracelular nas diversas fases do ciclo celular. Várias semanas após as culturas de fibroblastos humanos atingirem a senescência, os níveis de p21 diminuem gradualmente, conforme os níveis de p16 aumentam.^(18,19)^ Esses achados, e o fato de que o p21 não é induzido em células senescentes, deficientes em p53, e que têm tempo de vida replicativa prolongado,^(20,21)^ sugerem que o p53 inicia a inibição dessa replicação celular, pelo menos em parte, induzindo p21. O aumento subsequente em p16, então, age para manter a parada do crescimento celular, levando à senescência. Assim, mesmo sendo o p21 um marcador de envelhecimento celular, seu monitoramento termina por ser mais complexo e fora do escopo desse nosso estudo. Por outro lado, a observação dos níveis de p21 por citometria de fluxo, empregando também anticorpos que medem o grau de fosforilação da proteína, pode tornar o monitoramento da progressão da senescência celular com p21 de utilidade para o pesquisador.

Há limitações claras em nosso estudo, pois foram testadas apenas quatro amostras de um único tipo de tecido e seria importante compará-las com outras CTM, por exemplo, de medula óssea e de tecido adiposo, para validar essa estratégia muito prática de avaliar a progressão da senescência nas culturas. Tradicionalmente, as células têm sua validação em termos de qualidade, marcadores celulares e capacidade proliferativa feita em passagens baixas (passagens 4 ou 5), e, nas fases finais prévias à aplicação terapêutica, são feitos apenas os controles microbiológico e citogenético. O grau de senescência, ou seja, a capacidade de proliferação das células não é avaliada antes da aplicação terapêutica. Assim, acreditamos ser possível fazer o seguimento do aumento intracelular de p16 nas etapas de expansão das culturas com vistas à terapia celular. Esse seguimento pode ser útil para avaliar melhor a efetividade das células do doador, que, ao lado dos fatores inerentes aos receptores, como a idade e o tipo e gravidade da doença de base, impactam no sucesso da terapia com CTM.

## CONCLUSÃO

O seguimento de culturas celulares, por meio da imunofluorescência indireta de p16, permite identificar as culturas de células-tronco mesenquimais em risco de atingirem a senescência replicativa.
